# Painful vision loss during air travel after vitrectomy with air tamponade: a case report

**DOI:** 10.1186/s13256-024-05017-w

**Published:** 2025-01-10

**Authors:** Jessica Emrich, Peter Charbel Issa, Carmen Baumann

**Affiliations:** Department of Ophthalmology, TUM University Hospital, School of Medicine and Health, Technical University of Munich (TUM), Ismaninger Straße 22, 81675 Munich, Germany

**Keywords:** Air tamponade, Air travel, Central retinal artery occlusion, Intraocular pressure, Vitrectomy

## Abstract

**Background:**

While the potentially hazardous effects of intraocular perfluorocarbon gases during air travel have been recognized, the equivalent risk of intraocular air tamponade is less known and has, to the best of our knowledge, not been reported yet.

**Case presentation:**

A 52-year-old white female experienced a complete loss of vision and pain in her left eye during air travel following pars plana vitrectomy with air tamponade. Clinical and multimodal imaging findings only a few hours after emergency landing indicated a transient central retinal artery occlusion due to a significant increase in intraocular pressure during the flight.

**Conclusion:**

Air travel, even with a relatively small volume of residual air tamponade, can lead to potentially serious complications.

## Background

Perfluorocarbon gases and air are frequently used as intraocular tamponades following pars plana vitrectomy (PPV) [1]. In the event of a change in atmospheric pressure, for instance during air travel, a potentially hazardous effect of perfluorocarbon gases on retinal perfusion and vision, due to a sharp rise in intraocular pressure (IOP), has been reported [1–4]. However, an equivalent risk associated with intraocular air tamponade is less recognized.

## Case presentation

**Fig. 1:**
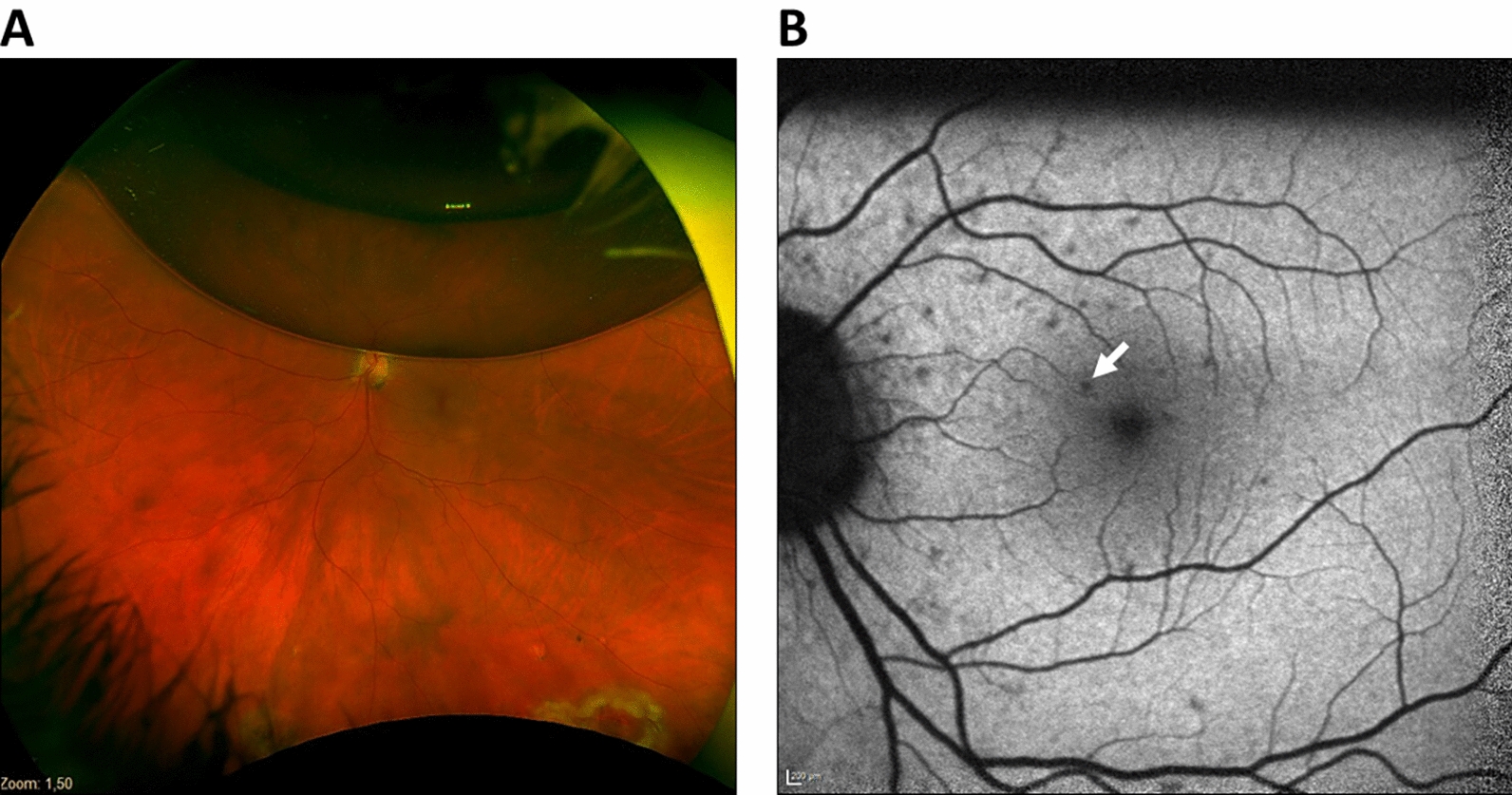
**A** Optos image of the left eye shows an approximately 30–40% air fill of the vitreous cavity. **B** Fundus autofluorescence image of the left eye shows small punctate hypofluorescent lesions at the posterior pole (arrow)

**Fig. 2:**
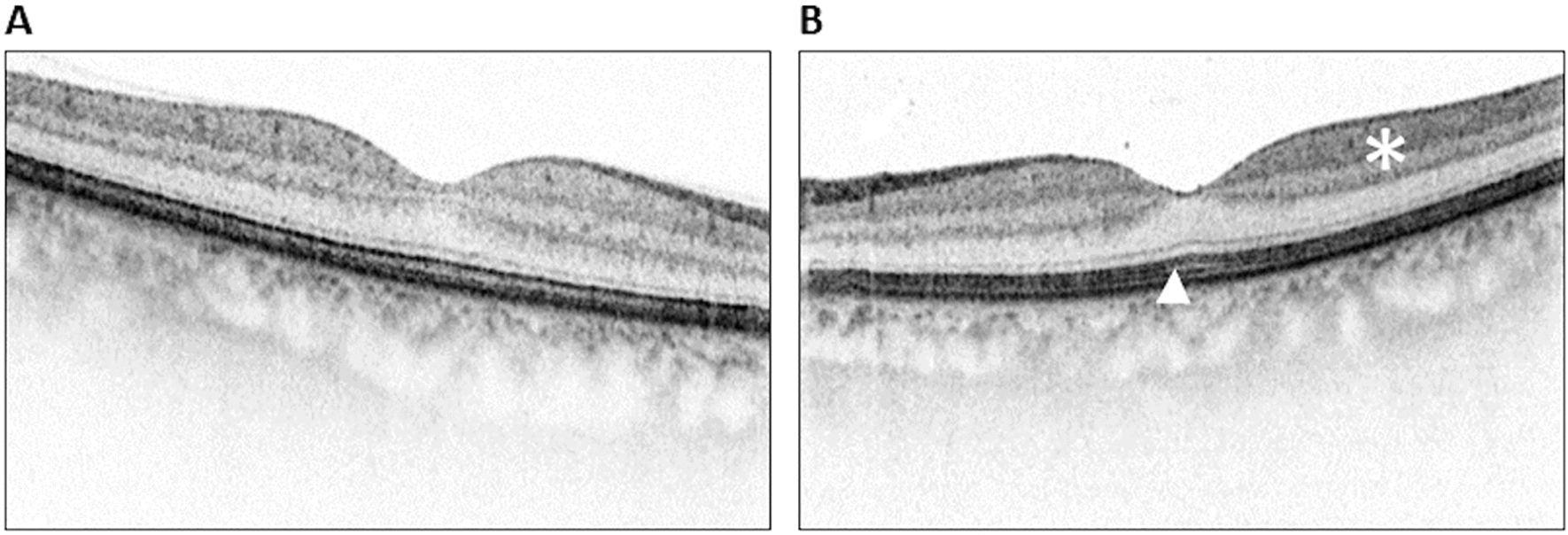
Optical coherence tomography scans of the macula. Compared with the fellow eye (**A**) on the left eye (**B**), the inner retinal layers appear slightly hyper-reflective (asterisk), and the ellipsoid zone seems mildly less well-demarcated (arrowhead)

Herein, we report the case of a 52-year-old white woman who boarded a flight four days after left PPV with air tamponade. About 15 minutes after takeoff, she experienced severe left-sided headache and complete loss of vision in the left eye (LE). Both improved during descent of the aircraft and had resolved after emergency landing about 1 hour after onset. After immediate transfer to our hospital, LE best-corrected visual acuity (BCVA) was 0.1 logMAR, and intraocular pressure (IOP) was 18 mmHg. Clinical examination revealed regular postoperative findings, with an approximately 30–40% air-filled vitreous cavity (Fig. 1A). However, fundus autofluorescence imaging of the LE showed small punctate hypofluorescent lesions at the posterior pole (Fig. 1B), and optical coherence tomography (OCT) imaging demonstrated a slightly increased reflectivity of the inner retinal layers, as well as a slightly less well-demarcated ellipsoid zone compared with the fellow eye (Fig. 2), which have previously only been reported in animal models [5]. Examination of the right eye (RE) was unremarkable. At 2-month follow-up, BCVA in the LE had improved to −0.1 logMAR, and OCT images of the macula and the peripapillary retinal nerve fiber layer (RNFL) and a 24–2 visual field (VF) test were normal.

## Discussion and conclusion

The severe left-sided pain and the complete loss of vision, along with subsequent hyper-reflectivity of the inner retinal layers on OCT, are consistent with a transient central retinal artery occlusion (CRAO) caused by a rapid increase in IOP, due to the expansion of the air bubble as atmospheric pressure decreases [2, 3]. While such scenarios have only been reported for gas-filled eyes [1, 4], the same principle also applies to air-filled eyes (Boyle’s law, the product of pressure and volume of ideal gases is constant) [1, 3].

The magnitude of the IOP increase and the resulting damage depend on additional factors beyond the tamponade volume, including cabin pressure (which varies between different airplane models), flight altitude and the speed at which flight altitude is reached, altitude of the departure and destination airports, flight duration, and the preoperative status of the eye and its IOP [2, 4]. Compensating mechanisms such as choroidal compression, scleral expansion, and increased aqueous humor outflow are only effective when changes in atmospheric pressure are small and/or slow [4]. Furthermore, such mechanisms might be impaired in patients with preexisting optic nerve or vascular disease, increasing their vulnerability to pressure-related damages. For example, while our patient did not demonstrate any abnormalities on RNFL-OCT and VF test, sectorial-decreased RNFL thickness, with a corresponding VF defect, has been reported in a 64-year-old man with glaucoma, 2 weeks after he took a flight with 10% C_3_F_8_ gas filling [1].

The only effective therapeutic intervention is reduction in flight altitude [4]. Prophylactic administration of IOP-lowering medication is insufficient [2].

There are inconsistent recommendations in the literature regarding the gas volume that can be tolerated during air travel [1–4]. However, such general statements are unreliable, owing to the numerous eye-specific and flight-specific factors that determine the magnitude of the IOP increase and the resulting damage. Air travel, even with a relatively small volume of residual air tamponade, can lead to potentially serious complications.

## Data Availability

Not applicable.
